# A dual phosphorylation switch controls 14-3-3-dependent cell surface expression of TASK-1

**DOI:** 10.1242/jcs.180182

**Published:** 2016-02-15

**Authors:** Markus Kilisch, Olga Lytovchenko, Eric C. Arakel, Daniela Bertinetti, Blanche Schwappach

**Affiliations:** 1 Department of Molecular Biology, Universitätsmedizin Göttingen, Humboldtallee 23, Göttingen 37073, Germany; 2 Department of Biochemistry, University of Kassel, Kassel 34132, Germany; 3 Max-Planck Institute for Biophysical Chemistry, Göttingen 37077, Germany

**Keywords:** 14-3-3 protein, Endoplasmic reticulum, Golgi, Membrane trafficking, Phosphorylation, Two-pore-domain K^+^ channel, COPI, Protein kinase A, TASK-1

## Abstract

The transport of the K^+^ channels TASK-1 and TASK-3 (also known as KCNK3 and KCNK9, respectively) to the cell surface is controlled by the binding of 14-3-3 proteins to a trafficking control region at the extreme C-terminus of the channels. The current model proposes that phosphorylation-dependent binding of 14-3-3 sterically masks a COPI-binding motif. However, the direct effects of phosphorylation on COPI binding and on the binding parameters of 14-3-3 isoforms are still unknown. We find that phosphorylation of the trafficking control region prevents COPI binding even in the absence of 14-3-3, and we present a quantitative analysis of the binding of all human 14-3-3 isoforms to the trafficking control regions of TASK-1 and TASK-3. Surprisingly, the affinities of 14-3-3 proteins for TASK-1 are two orders of magnitude lower than for TASK-3. Furthermore, we find that phosphorylation of a second serine residue in the C-terminus of TASK-1 inhibits 14-3-3 binding. Thus, phosphorylation of the trafficking control region can stimulate or inhibit transport of TASK-1 to the cell surface depending on the target serine residue. Our findings indicate that control of TASK-1 trafficking by COPI, kinases, phosphatases and 14-3-3 proteins is highly dynamic.

## Abstract (precis)

**Summary:** Phosphorylation of a previously neglected serine residue in the trafficking control region of TASK-1 interferes with the binding of trafficking machinery and enables complex regulation by physiological stimuli.

## INTRODUCTION

Two-pore-domain K^+^ (K2P) channels are dimeric membrane proteins that function at the cell surface and contribute to the electrical properties of many different cell types (Renigunta et al., 2015). It is well established that two members of the K2P-channel family – i.e. TASK-1 and TASK-3 (also known as KCNK3 and KCNK9, respectively) – require phosphorylation-dependent high-affinity binding of 14-3-3 proteins to their distal C-terminus in order to reach the cell surface (O'Kelly et al., 2002; Rajan et al., 2002; Smith et al., 2011; Kilisch et al., 2015). 14-3-3 proteins are small adaptor proteins with no specific enzyme activity; they can modulate the function of many ‘client’ proteins. The distal C-terminus of both channels functions as a trafficking control region; it contains a binding motif for the COPI vesicle coat and an overlapping mode III 14-3-3-binding motif [R-x-x-pS/pT-x-COOH, where ‘p’ denotes a phosphorylated residue and ‘x’ any amino acid (Coblitz et al., 2005)]. In the classic model of TASK trafficking, interaction with COPI retains TASK channels in the early secretory pathway until phosphorylation of the distal C-terminus allows binding of 14-3-3, which sterically prevents COPI from accessing the trafficking control region. Consequently, the phosphorylated channel exits the Golgi and is expressed at the cell surface. Based on studies using *in vitro* phosphorylation, on mutagenesis or application of specific inhibitors, protein kinase A (cAMP-dependent protein kinase, PKA) has emerged as the kinase that is most likely to be responsible for phosphorylating TASK C-termini (Mant et al., 2011). However, the direct effect of phosphorylation on COPI binding has not yet been determined, and the protein–protein interactions involved in TASK trafficking control have not been assessed quantitatively. Most studies treat the seven 14-3-3 proteins (encoded by seven distinct genes but commonly termed isoforms in the field) as one entity, and little is known about the significance of individual 14-3-3 proteins (denoted with Greek letters as β, γ, η, ε, σ, τ and ζ) in modulating the function of specific 14-3-3 clients.

The observation that the intracellular trafficking of TASK channels depends on phosphorylation and interaction with 14-3-3 proteins suggests that the surface expression of TASK channels might be regulated by protein kinases and phosphatases. This aspect is poorly understood because studies in heterologous systems have focused on the fundamental prerequisites for cell surface expression rather than on its modulation by signal transduction. However, the cell surface expression of many channels is highly regulated under different physiological conditions. We have recently shown that the COPI-binding motif, which prevents the cell surface expression of ATP-sensitive K^+^ channels, can be phosphorylated and thus inactivated upon β-adrenergic stimulation in cardiac myocytes (Arakel et al., 2014). Here, we have used an array of wild-type and mutated TASK distal C-termini, and all seven mammalian 14-3-3 proteins to systematically and quantitatively delineate the molecular events that determine the role of the TASK trafficking control region in regulated cell surface expression.

## RESULTS

### Affinity of 14-3-3 for the TASK-1 C-terminus is substantially lower than that for the TASK-3 C-terminus

**Fig. 1.**
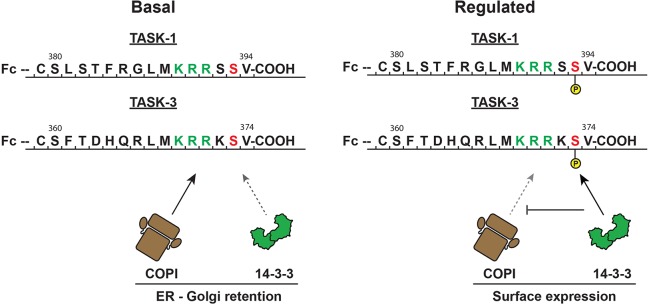
**Peptides and proteins used in this study.** The last 15 amino acids of human TASK-1 and TASK-3 C-termini contain a minimal trafficking control region comprising a COPI recognition motif (green), a PKA target site (red) and a mode III 14-3-3-binding motif (-K-R-R-K/S-pS-V-COOH, where ‘p’ denotes a phosphorylated residue). Note the presence of a serine residue (S392) preceding the conserved serine residue of the mode III 14-3-3-binding motif in TASK-1 (S393) but not TASK-3 (S373). Fluorescein-labeled peptides comprising the TASK C-termini were used for the determination of equilibrium binding constants by using fluorescence polarization.

Current insight into COPI and 14-3-3 binding to the trafficking control region of the TASK C-terminus is qualitative (O'Kelly et al., 2002; Rajan et al., 2002; O'Kelly and Goldstein, 2008; Zuzarte et al., 2009). The equilibrium dissociation constant (*K*_d_) for the binding of 14-3-3σ to a hexapeptide corresponding to the C-terminus of TASK-3 that included phosphorylated residue S373 has been determined previously (4.1±0.8 µM; Anders et al., 2013), but it is not known how the parameters of 14-3-3 binding compare between TASK-1 and TASK-3, or between the different 14-3-3 isoforms. To determine them systematically, we chose fluorescence polarization titration – a solution-based method suited to measuring the binding affinity between one 14-3-3 ligand-binding groove and the TASK-derived peptide (Fig. 1A,B). The use of synthetic fluorescein-labeled peptides is particularly useful for studying the C-terminus of TASK-1 because the phosphorylation status of the two serine residues (S392 and S393) present in its sequence can be controlled individually. All seven human 14-3-3 isoforms were affinity purified as fusions to the maltose binding protein (MBP). After cleavage of the MBP tag, dimeric 14-3-3 proteins were purified by using size exclusion chromatography (Fig. S1).

**Fig. 2.**
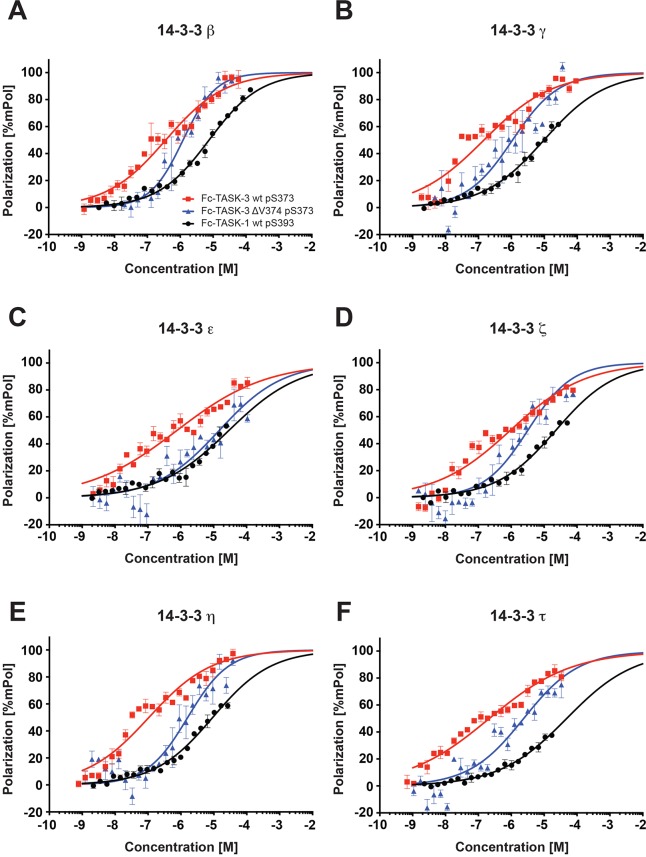
**TASK-1 C-terminus binds to 14-3-3 proteins with 10- to 100-fold lower affinity than the TASK-3 C-terminus.** Fluorescence polarization titration of fluorescein-labeled TASK-1 pS393 (black), TASK-3 pS373 (red) and TASK-3 pS373 ΔV374 (blue) peptide (10 nM) with increasing concentrations of the indicated 14-3-3 isoform. Fluorescein was excited at 485 nm, and fluorescence polarization was monitored at 535 nm. A monophasic fit was used to fit the data (Goodness-of-fit: R^2^ from 0.76 to 0.98). Equilibrium constants determined from the fit are listed in Table 1. No binding was detectable for the unphosphorylated TASK C-terminal peptides. The assay was repeated at least six times for each data point with two batches of independently purified 14-3-3 proteins. Error bars depict s.e.m. (A) 14-3-3β, (B) 14-3-3γ, (C) 14-3-3ε, (D) 14-3-3ζ, (E) 14-3-3η and (F) 14-3-3τ.

TASK-1 or TASK-3 C-terminal fluorescent peptides (10 nM), comprising the last 15 amino acids of the respective C-termini, were titrated with concentrations of the 14-3-3 proteins ranging from 1 nM to 120 µM (Fig. 2), and the equilibrium dissociation constants of 14-3-3 proteins were calculated for TASK-1 phosphorylated at S393 (TASK-1 pS393; the conserved serine residue of the mode III 14-3-3-binding motif) and for TASK-3 phosphorylated at S373 (TASK-3 pS373) (Table 1). They ranged from 7.5±0.14 µM (mean±s.e.m.; 14-3-3β) to 49.4±2 µM (14-3-3τ) for TASK-1, whereas dissociation constants for TASK-3 ranged from 110±10 nM (14-3-3η) to 3.6±0.44 µM (14-3-3σ). The latter value matches the result determined previously by Anders et al. (2013) for the TASK-3 C-terminal hexapeptide using isothermal titration calorimetry. The dissociation constants of the seven mammalian 14-3-3 isoforms for the same TASK C-terminal peptide differed up to sevenfold for TASK-1-derived peptides and up to 30-fold for TASK-3-derived peptides. For both TASK C-termini, 14-3-3γ and 14-3-3η bound with high affinity, whereas 14-3-3σ bound with low affinity. No binding of any 14-3-3 isoform to any TASK C-terminal peptide was observed when the peptides were not phosphorylated.

**Table 1.**
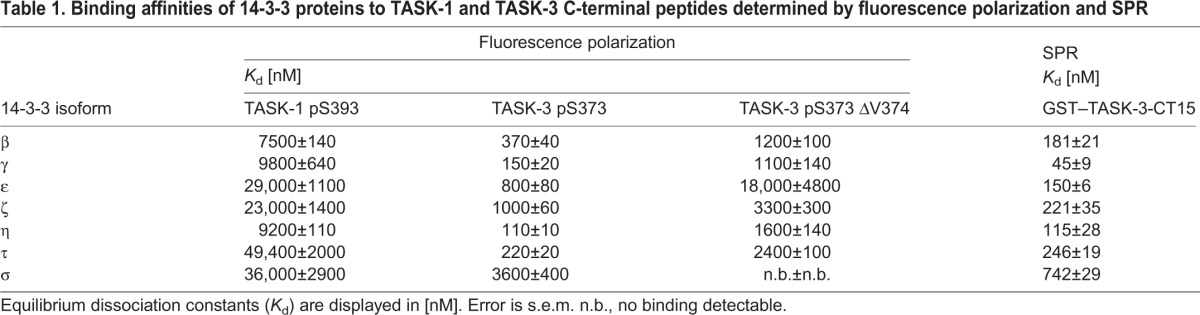
**Binding affinities of 14-3-3 proteins to TASK-1 and TASK-3 C-terminal peptides determined by fluorescence polarization and SPR**

**Fig. 3.**
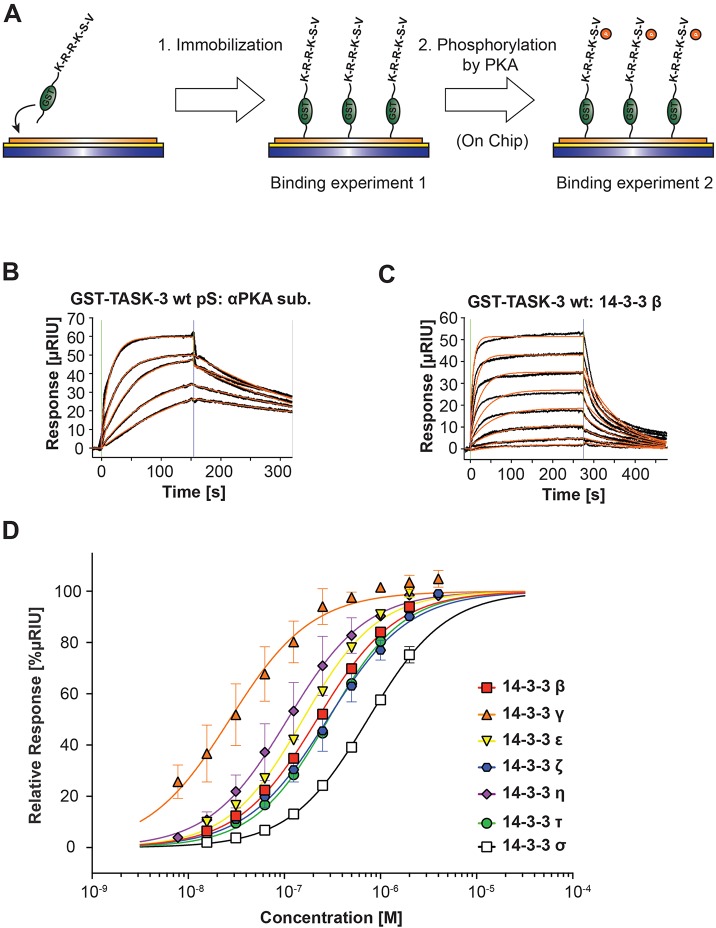
**Affinities of the seven mammalian 14-3-3 isoforms for the phosphorylated TASK-3 C-terminus obtained by surface plasmon resonance are consistent with values obtained using fluorescence polarization.** (A) Schematic illustration of the surface plasmon resonance (SPR) set-up used to monitor 14-3-3 binding to immobilized TASK-3 C-termini fused to GST and phosphorylated on the carboxymethyldextran sensor chip. (B) An antibody against PKA-phosphorylated substrates was used to confirm on-chip phosphorylation of the TASK-3 C-termini. (C) Representative family of SPR sensograms reflecting the association and dissociation phases of 14-3-3β and 14-3-3σ binding to immobilized phosphorylated TASK-3 C-termini. No binding was observed before phosphorylation of the immobilized GST fusion protein. (D) Dose-response curve for the respective pairs of GST–TASK-3 C-terminal fusion proteins and the individual 14-3-3 proteins with SPR analysis. Data were analyzed with a sigmoidal dose-response curve fit (Goodness-of-fit: R^2^ from 0.96 to 0.99). Equilibrium constants determined from the fit are listed in Table 1. The assay was repeated at least six times for each data point with two batches of independently purified 14-3-3 proteins. Error bars depict s.e.m.

In addition to fluorescence polarization, we used surface plasmon resonance (SPR) as a second, complementary approach to quantify the binding parameters for the TASK-3 C-terminus (Fig. 3, Table 1). A glutathione S-transferase (GST)-tagged TASK-3 C-terminal 15 residues fusion protein (GST–TASK-3) was expressed in and purified from *Escherichia* *coli* (and hence was not phosphorylated). We developed an on-chip phosphorylation protocol (Fig. 3A) where we first captured the GST fusion proteins on the chip surface with an antibody against GST (which was crosslinked at a high density to the metal surface) and then phosphorylated GST–TASK-3 with recombinantly expressed PKA catalytic subunit (also known as PRKACA; Knape et al., 2015). We verified efficient phosphorylation of the fusion proteins by examining the binding parameters of a phospho-specific antibody that recognizes a phosphorylated PKA target motif to the PKA-treated TASK-3 C-terminus (Fig. 3B). We observed high-affinity binding with an equilibrium dissociation constant of 4.5±0.6 nM. Consistent with the results of fluorescence polarization titration, no binding of 14-3-3 proteins was observed prior to on-chip phosphorylation, whereas all 14-3-3 isoforms did bind upon phosphorylation of the TASK-3 C-terminus (Fig. 3C) with equilibrium dissociation constants (Fig. 3D, Table 1) between 45±9 nM (14-3-3γ) to 742±29 nM (14-3-3σ). Depending on the 14-3-3 isoform, these values were very similar (14-3-3η and 14-3-3τ) or differed two- (14-3-3β) to fivefold (14-3-3ε, 14-3-3ζ and 14-3-3σ) from the values determined by fluorescence polarization (Fig. 2, Table 1). Importantly, 14-3-3γ and 14-3-3η displayed the same high-affinity binding as that observed in the fluorescence polarization assay. We conclude that the two methods yield comparable parameters for 14-3-3 binding to the TASK-3 pS373 C-terminal peptide. The differences in absolute values might be due to the fact that the presentation of the phosphorylated C-termini is different in the two methods (soluble peptide compared to immobilized GST fusion protein).

### Deletion of the C-terminal valine residue in the TASK-3 C-terminus does not abolish 14-3-3 binding

Based on yeast two-hybrid and qualitative pull-down assays, it has been proposed that deleting the C-terminal valine residue of the mode III 14-3-3-binding motif abolishes 14-3-3 binding (O'Kelly et al., 2002; Rajan et al., 2002). To test this hypothesis, we measured the affinity of all seven 14-3-3 isoforms for the TASK-3 C-terminal peptide containing phosphorylated S373, but lacking the C-terminal valine (V374), by fluorescence polarization titration (Fig. 2, Table 1). Indeed, the interaction could no longer be measured for 14-3-3σ, but the affinities remained in the low micromolar range for 14-3-3β, 14-3-3γ, 14-3-3ζ, 14-3-3η and 14-3-3τ. The C-terminal deletion reduced the affinity between three- (14-3-3β) and 23-fold (14-3-3 epsilon), but the absolute values for the equilibrium dissociation constants were lower than those observed for the TASK-1 C-terminus with both pS393 and V394 (Fig. 2, Table 1). Hence, it appears that the reported lack of surface expression observed in TASK-3 reporter constructs upon deletion of the C-terminal valine (O'Kelly et al., 2002; Rajan et al., 2002) cannot be fully explained by reduced 14-3-3 binding.

**Fig. 4.**
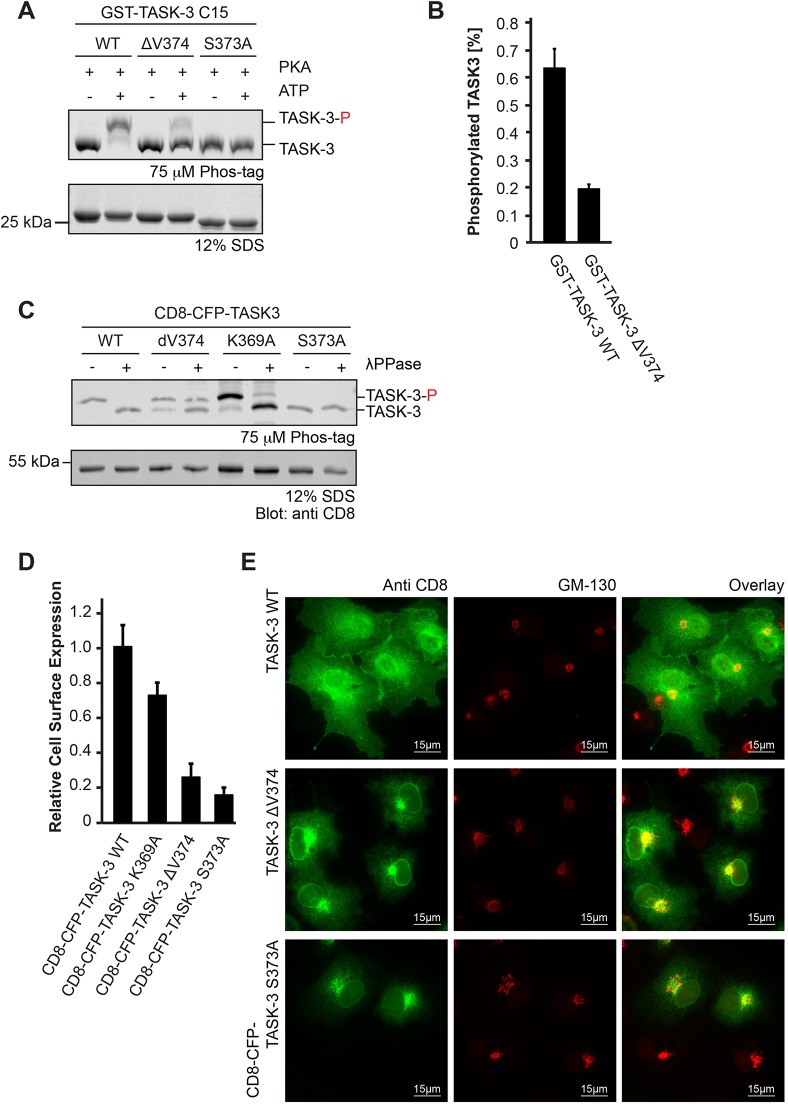
**Reduced 14-3-3 binding affinity does not explain the lack of cell surface transport of the TASK-3 ΔV mutant.** (A) *In vitro* phosphorylation of recombinant GST–TASK-3 (15 C-terminal residues) wild-type (WT), GST–TASK-3 ΔV374 and GST–TASK-3 S373A fusion proteins with the PKA catalytic subunit. Samples were resolved on an SDS-PAGE gel containing 75 µM Phostag reagent and 75 µM MnCl_2_, which retards the migration of proteins by chelating phosphorylated amino acid side chains. The Coomassie-stained gel is representative of six independent experiments. TASK-3-P, phosphorylated TASK-3. (B) Bar diagram of the relative amount of phosphorylated TASK-3 trafficking control region constructs shown in A. *n*=6, error bars depict s.e.m. (C) *In vivo* phosphorylation status of the indicated CD8–CFP reporter proteins, as reflected by migration in Phostag SDS-PAGE gels. λPPase, λ phosphatase. The blot is representative of 11 independent transfections. (D) Flow cytometry assay to determine the effect of the indicated TASK C-termini on the cell surface expression of a CD8–CFP reporter protein. Cells were transfected with the same series of constructs in three independent experiments. For each experiment, 10,000 cells per construct were analyzed. The mean of the CD8 signal was expressed as a ratio with the mean of the CFP signal in CFP-positive cells and normalized to that of the wild-type TASK-3 construct. Error bars depict s.e.m. (E) Subcellular localization of CD8–CFP–TASK-3 ΔV374 is indistinguishable from that of CD8–CFP–TASK-3 S373A. Constructs were transfected in three independent experiments and ca. 100 cells per transfection were analyzed by indirect immunostaining against the indicated antigens.

To further clarify this point, we studied the phosphorylation of GST fusion proteins of the corresponding C-terminal TASK-3 peptides by using the recombinant PKA catalytic subunit *in vitro* (Fig. 4A), and we found that the efficiency of phosphorylation was reduced about threefold for the GST–TASK-3 ΔV374 C-terminus compared to that for the wild type (Fig. 4B). Analysis of the phosphorylation state of S373 in TASK-3 lacking the terminal valine by transiently transfecting a reporter construct that included the extracellular domain of CD8 (CD8–CFP–TASK-3 ΔV374) into COS-7 cells and subsequent SDS-PAGE analysis with gels containing a phospho-chelating agent (Phostag) also revealed that the efficiency of S372 phosphorylation was decreased in the absence of the C-terminal valine. The reporter proteins exposing the wild-type TASK-3 C-terminal 15 residues or a mutant, in which the COPI-dependent ER retention–retrieval motif (KRR) was disrupted (K369A), migrated slower than the S373A mutant, implying that both the wild-type TASK-3 and the K369A mutant were phosphorylated. Consistent with this interpretation, treatment with phosphatase altered the migratory behavior of the reporter proteins to match that of the S373A mutant. In contrast to the wild type and to the K369A variant, we observed substantial amounts of unphosphorylated CD8–CFP–TASK-3 ΔV374 reporter protein prior to phosphatase treatment. These findings suggest that truncation of the mode III 14-3-3-binding motif affects the efficiency of phosphorylation by PKA *in vivo* and might also improve access for cellular phosphatases.

To assess the functional consequences of phosphorylation on cell surface expression of the reporter protein, we employed flow cytometry (Fig. 4C). In this assay, transfected cells were identified using the CFP fluorescence signal, and the relative cell surface expression was determined by staining an extracellular epitope of CD8 with a specific antibody. We determined the relative cell surface expression of wild-type CD8–CFP–TASK-3 as well as that of the ΔV374 and S373A mutants, and we found that the C-terminal truncation variant displayed moderately higher cell surface expression levels than the non-phosphorylatable S373A mutant. We also assessed the steady-state localization of the bulk of the CD8–CFP fusion proteins with indirect immunostaining (Fig. 4D; Fig. S2) and observed a very similar pattern for both, CD8–CFP–TASK-3 ΔV374 and CD8–CFP–TASK-3 S373A. As reported previously (Zuzarte et al., 2009), proteins unable to recruit 14-3-3, which is required to counteract the COPI-binding motif present in the C-terminus of TASK proteins, accumulated in a punctate perinuclear COPI-positive structure (Fig. S2) in the vicinity of a marker of the Golgi compartment (GM130, also known as GOLGA2; Fig. 4D). We conclude that in the mutant TASK-3 ΔV374, less of the CD8–CFP reporter fusion protein is phosphorylated and that the phosphorylated form binds to 14-3-3 proteins with lower affinity than wild-type TASK-3 but as strongly as TASK-1 pS393. The simplest interpretation of these findings is that strongly reduced cell surface expression of C-terminally truncated TASK-3 is attributable to the combination of inefficient phosphorylation and a moderately reduced 14-3-3 binding affinity.

### Phosphorylated S392 in the TASK-1 C-terminus inhibits 14-3-3 binding

**Fig. 5.**
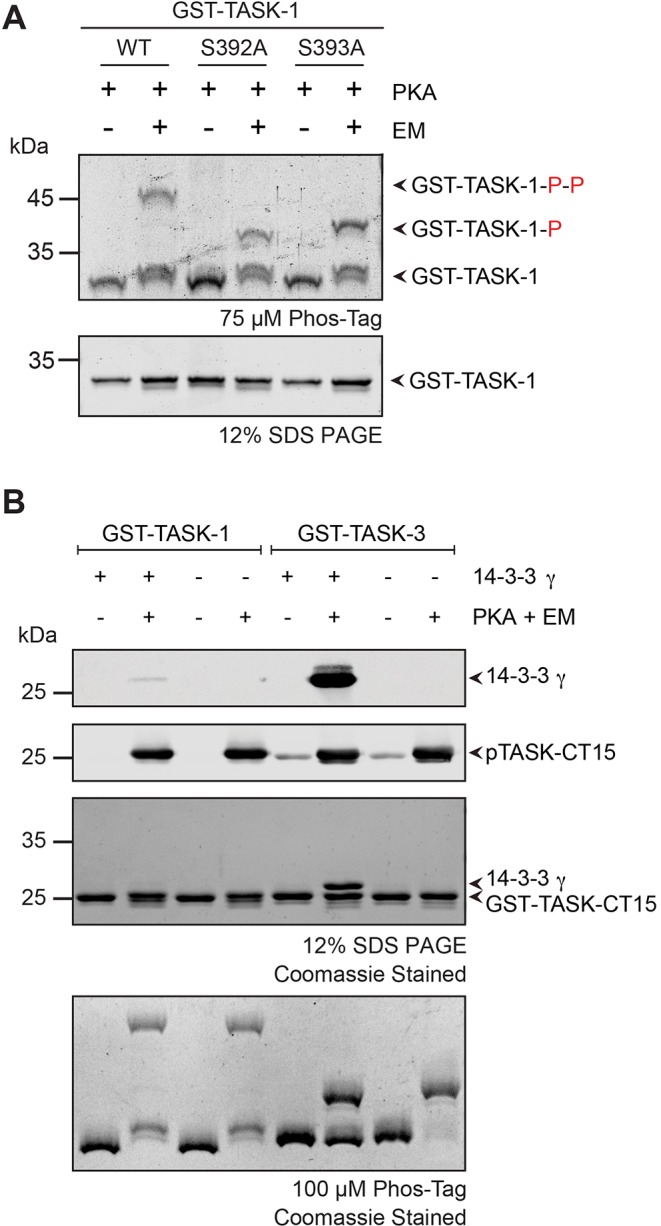
**Phosphorylation of S392 in TASK-1 inhibits 14-3-3 binding.** (A) *In vitro* phosphorylation of recombinant GST–TASK-1 wild-type, GST–TASK-1 S392A and GST–TASK-1 S393A fusion proteins with PKA catalytic subunit. Samples were resolved on an SDS-PAGE gel containing 75 µM Phostag reagent and 75 µM MnCl_2_. ‘P’ suffixes denote the number of phosphorylated residues. The gel is representative of three independent experiments. (B) 14-3-3 protein binding to the TASK-1 C-terminal trafficking control region is abolished by double phosphorylation. GST-TASK-1 or GST-TASK-3 C-terminal fusion proteins were *in vitro* phosphorylated using recombinant PKA catalytic subunit and an ATP regeneration system (energy mix; EM). Samples were resolved on SDS-PAGE gels (upper three panels) and on an SDS-PAGE gel containing 100 µM Phostag reagent and 100 µM MnCl_2_ (bottom). The upper two panels show western blots using antibodies against the indicated proteins, and the lower two panels show Coomassie-stained gels. TASK-CT15, the last 15 amino acids of the TASK C-terminus.

Mant et al. (2011) report that both serine residues present in the TASK-1 C-terminus can be phosphorylated *in vitro* by the recombinant catalytic subunit of PKA. We confirmed this result (Fig. 5A) using GST fusion proteins of the 15 C-terminal residues of TASK-1, in which either one or both of the residues S392 or S393 were phosphorylated. Next, using fluorescence polarization titration, we determined how phosphorylation of S392 affects the affinity of all seven mammalian 14-3-3 proteins for the TASK-1 C-terminus – either when phosphorylated alone or in combination with phosphorylation of S393 (Table S1). In each case, the presence of phosphorylated S392 significantly reduced the affinity of all 14-3-3 proteins for the TASK-1-derived peptide, which bound to 14-3-3 with dissociation constants in the low micromolar range (Table S1). In contrast, the *K*_d_ values obtained for TASK-1 pS392 were ∼100 µM, whereas those for the doubly phosphorylated peptide were in the high micromolar range. The strongly reduced 14-3-3 binding affinity of the doubly phosphorylated peptide was confirmed with a qualitative pull-down assay (Fig. 5B) using the GST fusion proteins that exposed the TASK-1 or TASK-3 C-terminal 15 residues. Both constructs were efficiently phosphorylated by recombinant PKA, as demonstrated with Phostag SDS PAGE analysis (lowest panel) or by blotting with an antiserum against phospho-TASK C-termini that had been generated in our laboratory (second panel from top). Although recombinant 14-3-3γ was efficiently pulled down using TASK-3 p373 (top panel and Coomassie-stained panels at the bottom), only a very faint band was observed for the doubly phosphorylated TASK-1 C-terminus. This indicates that the *K*_d_ of ∼200 µM measured for this interaction (Table S1) was too high to result in substantial co-purification of 14-3-3γ in this assay. Because all *K*_d_ values determined for TASK-1 pS392 and the doubly phosphorylated protein (at S392 and S393) were substantially higher than those measured for TASK-1 pS393 (Table S1), we conclude that phosphorylation of S392 cannot substitute for phosphorylated S393 in creating a high-affinity 14-3-3 binding site. On the contrary, our results are consistent with an inhibitory role for phosphorylation of S392 with respect to 14-3-3-mediated binding.

**Fig. 6.**
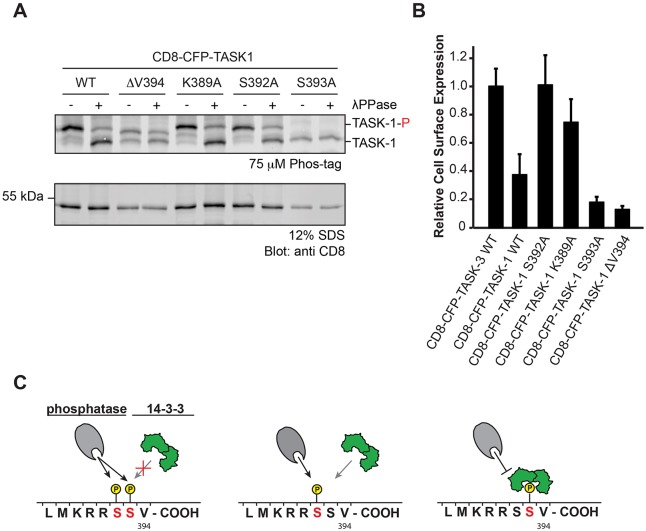
**Phosphorylation of S392 in TASK-1 reduces cell surface expression.** (A) *In vivo* phosphorylation status of the CD8 reporter protein as reflected by migration in Phostag SDS-PAGE gels. The blot is representative of seven independent transfections. TASK-1-P, phosphorylated TASK-1. (B) Flow cytometry assay to determine the effect of replacing either S392 or S393 in the TASK-1 C-terminus on the cell surface expression of the indicated CD8–CFP reporter proteins. Cells were transfected with the same series of constructs in three independent experiments. For each experiment, 10,000 cells per construct were analyzed. The mean of the CD8 signal is expressed as a ratio with the mean of the CFP signal in CFP-positive cells and normalized to that for the wild-type TASK-3 construct. Error bars depict s.e.m. (C) Model of the effect of phosphorylation of S392 or S393, or both on 14-3-3 binding and on the access of phosphatases. Note that only the population of TASK-1 reporter proteins phosphorylated on only S393 is expected to be protected from phosphatase action.

To test this hypothesis, we assessed the effect of mutating S392 on the steady-state phosphorylation and on the cell surface expression of CD8–CFP–TASK-1 in transfected cells (Fig. 6). In line with the results obtained with CD8–CFP–TASK-3 in Fig. 4B, Phostag gel electrophoresis analysis confirmed that the reporter fusion protein displaying the wild-type TASK-1 C-terminus was indeed phosphorylated (Fig. 6A). Interestingly, the wild-type, the K389A, the ΔV394 and the S392A constructs migrated in an identical manner, indicating that only one of the serine residues is efficiently phosphorylated or protected from dephosphorylation (Fig. 6B). It is generally assumed that binding of 14-3-3 proteins to a phosphorylated 14-3-3 target site acts to protect the phosphorylated side-chain from dephosphorylation by phosphatases (Kagan et al., 2002). Thus, TASK-1 C-termini exposing phosphorylated S392 or phosphorylated S392 and S393 residues will be efficiently dephosphorylated because they bind to 14-3-3 proteins with lower affinity (Table S1), whereas phosphorylated S393 should be protected by bound 14-3-3 proteins (Fig. 6A). In agreement with this hypothesis, for the S393A mutant, we observed only a minor band reflecting the phosphorylated species. We conclude that S392 can be phosphorylated by the catalytic subunit of PKA (Fig. 5), but our results suggest that either phosphorylation is inefficient or that this residue is efficiently dephosphorylated in COS-7 cells.

### S392 reduces the cell surface expression of CD8–CFP–TASK-1

To test the functional consequences of S392 phosphorylation in transfected cells, we determined the relative cell surface expression of CD8–CFP–TASK-1 S392A using flow cytometry (Fig. 6B). Surprisingly, the surface expression of this construct was more than twofold higher than that of ‘wild-type’ CD8–CFP–TASK-1. In contrast, CD8–CFP–TASK-1 S393A did not reach the cell surface, most probably owing to its inability to bind to 14-3-3 proteins. This finding is consistent with the results obtained with the CD8–CFP–TASK-3 S373A mutant (Fig. 4C).

It has been reported previously that residue K389 in the trafficking control region is a crucial residue of the ER localization motif of TASK-1 (Zuzarte et al., 2009). Mutating the lysine residue to alanine induced the same twofold increase in CD8–CFP–TASK-1 surface expression as the S392A mutation. These observations are consistent with the notion that transient phosphorylation of S392 is sufficient to reduce the efficacy by which 14-3-3 proteins can remove the cargo protein from the part of the secretory pathway where COPI operates. In the absence of a functional COPI-interaction motif (Zuzarte et al., 2009), 14-3-3 protein binding to the TASK C-terminus is no longer required for cell surface expression. Hence, the corresponding construct K389A reaches the cell surface efficiently. For TASK-3, which displays high-affinity 14-3-3 binding and no second inhibitory serine, the corresponding K369A mutant is expressed at levels at the cell surface that are nearly equal to those of the TASK-3 wild-type construct (Fig. 4C). Hence, in the case of TASK-3, 14-3-3 efficiently displaces COPI. The reduced cell surface expression of the TASK-1 wild-type construct with respect to both the TASK-1 K389A and S392A constructs is consistent with the notion that both COPI and 14-3-3 proteins can access the trafficking control region in the steady state.

### Phosphorylation of TASK C-termini abolishes COPI binding

**Fig. 7.**
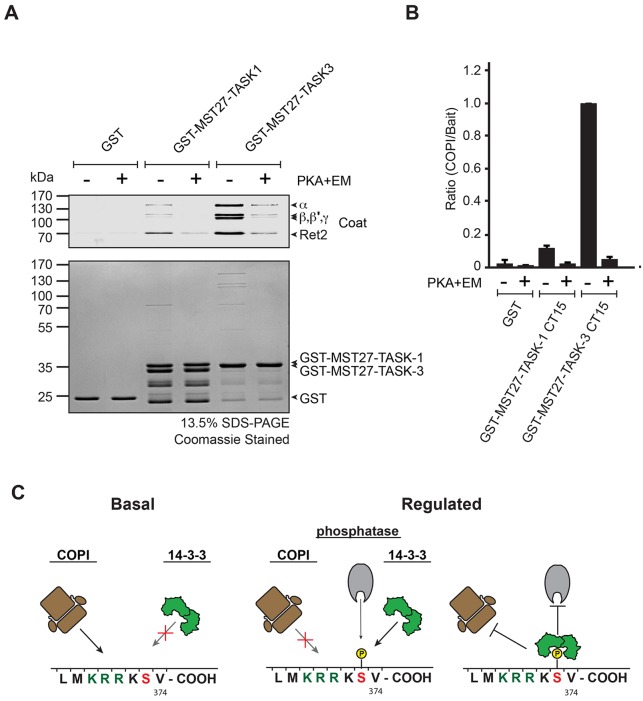
**Phosphorylation of TASK C-termini inhibits COPI binding.** (A) GST–Mst27–TASK-1 or GST–Mst27-TASK-3 fusion proteins comprising the last 15 amino acids of the C-termini (CT15) were *in vitro* phosphorylated using recombinant PKA catalytic subunit and an ATP regeneration system (energy mix; EM). Upper panel shows a western blot and detection with an anti-yeast COPI antiserum, lower panel shows a Coomassie-stained gel. (B) Quantification of three independent pull-down experiments similar to that depicted in A. Error bars depict s.e.m. (C) Model of the effect of phosphorylation of S373 in TASK-3 on the binding of COPI, access of phosphatases and 14-3-3 binding. Note that binding of COPI and 14-3-3 proteins is not competitive.

Although it is well established that phosphorylation of the mode III binding motif is a prerequisite for 14-3-3 binding to TASK C-termini, the effect of phosphorylation on COPI binding is unknown. Functionally, the COPI-binding motif was mapped in the context of TASK ΔV constructs because retention of these constructs from the cell surface reflects COPI activity. Pull-down assays to directly assess the physical interaction were performed with cellular lysates that also contained kinases, phosphatases and 14-3-3 proteins (O'Kelly et al., 2002; O'Kelly and Goldstein, 2008; Zuzarte et al., 2009). In these experiments, detection of the β-COP (also known as COPB1) subunit served as a proxy for binding of the heptameric COPI coat. To assess the physical interaction with COPI in the absence of additional binding partners (Fig. 7), we exploited our previous finding that the ER retention–retrieval signal of TASK-3 is also recognized in yeast (Zuzarte et al., 2009). We purified the COPI coat from yeast using tandem-affinity purification (TAP) of tagged β-COP (Yip and Walz, 2011) and tested its interaction with the TASK-1 and TASK-3 trafficking control region (15 C-terminal residues) presented on the cytoplasmic tail of the yeast protein Mst27, which lacked its KKXX signal and had been fused to GST, as described previously (Sandmann et al., 2003). This Mst27 linker increases the accessibility of the C-terminal peptide to the heptameric COPI coat and has been extensively characterized in COPI-binding experiments (Sandmann et al., 2003; Michelsen et al., 2007). We found that both immobilized GST–TASK-1 and GST–TASK-3 enriched COPI before but not after phosphorylation of the TASK tails (Fig. 7A). Quantification of three independent experiments showed that TASK-1 bound to COPI less efficiently than TASK-3 (Fig. 7B). We conclude that COPI ceases to interact with the phosphorylated trafficking control region of TASK-1 and TASK-3 channels even in the absence of 14-3-3 proteins. This implies that there is no direct competition between COPI and 14-3-3 binding (Fig. 7C). Instead, the respective binding equilibria exist between either the unphosphorylated (COPI) or phosphorylated (14-3-3) forms of the TASK C-terminus and hence are coupled through the action of kinases and phosphatases.

## DISCUSSION

The trafficking control region in the C-termini of the K2P channels TASK-1 and TASK-3 is thought to control the surface expression of the channels through the mutually exclusive binding of either the COPI vesicle coat, which mediates retrieval to the early secretory pathway, or of 14-3-3 proteins, which bind in a phosphorylation-dependent manner and protect the region from COPI binding (Smith et al., 2011). In essence, the trafficking control region is thought to function based on competitive binding events. However, our mechanistic understanding of this trafficking control region is far from complete. A full quantitative model of this competition and its outcome would require insight into the copy numbers of the cargo, COPI and of 14-3-3 proteins as well as the dissociation constants of the pertinent binding events. Furthermore, there are striking differences in the residues that flank the COPI-dependent retention–retrieval motif and the mode III 14-3-3-binding motif of TASK-1 and TASK-3; TASK-1 features a second serine that can be phosphorylated (S392) and that precedes the conserved serine residue of the mode III motif (S393). This serine residue is absent in TASK-3, where the corresponding residue immediately following the KRR retention–retrieval motif is lysine (K372). Here, we present evidence that this sequence difference between the TASK-1 and TASK-3 C-termini causes biologically relevant differences in COPI and 14-3-3 protein binding that might render TASK-1 more amenable to regulation.

We determined the binding parameters for all seven mammalian 14-3-3 isoforms to both TASK protein C-termini and to their variants (phosphorylated or non-phosphorylated peptides and mutants thereof). Interestingly, the highest affinity of the TASK-1-derived pS393 peptide to 14-3-3 was approximately two orders of magnitude lower than that of the TASK-3-derived peptide (the *K*_d_ value was ∼10 µM compared to 100 nM; Fig. 2, Table 1). This illustrates that the numerous pull-down and yeast two-hybrid experiments previously used to assess this interaction (O'Kelly et al., 2002; Rajan et al., 2002; Smith et al., 2011) effectively operate down to *K*_d_ values of 10 µM. However, owing to their qualitative nature, the yeast two-hybrid experiments obscure substantial affinity differences. We also showed that pull-down experiments cease to efficiently capture an interaction if the *K*_d_ is in the order of 200 µM (Table S1, Fig. 5) or higher.

The marked differences that we found between the affinities of TASK-1 or TASK-3 for 14-3-3 proteins are in excellent agreement with the structure (Anders et al., 2013) of this peptide bound to 14-3-3σ; K372 of TASK-3, which is centrally positioned in the trafficking control region and corresponds to a serine residue in TASK-1, forms hydrogen bonds with an aspartate and asparagine residue of the 14-3-3-binding groove. A phosphoserine residue in the position equivalent to K372 of TASK-3 is very likely to destabilize the interaction with 14-3-3 proteins, and this is exactly what we observed for TASK-1 pS392 and the doubly phosphorylated protein (at S392 and S393) (Fig. 5, Table S1). Because the exact binding site for the trafficking control region on COPI is unknown, we cannot interpret the relevance of K372 in TASK-3 with respect to the higher affinity of its trafficking control region to COPI when compared to that of TASK-1. The consensus of the COPI-binding motif requires basic residues in specific positions (Zuzarte et al., 2009). Hence, we speculate that the additional lysine residue in TASK-3 contributes to the high-affinity binding of COPI to the corresponding trafficking control region.

The differences between the C-termini of TASK-1 and TASK-3 in the affinities for COPI and 14-3-3 proteins were also found to be relevant in intact cells. We demonstrated that the CD8–CFP reporter comprising the exposed TASK-1 wild-type C-terminus reaches the cell surface less efficiently than the corresponding TASK-3 construct (Fig. 6). Mutation of S392 to alanine has the same effect as mutating K389 to alanine, which mutates the retention–retrieval motif (Zuzarte et al., 2009). We interpret this to be due to an increase in 14-3-3 protein affinity because S392, which results in strongly reduced 14-3-3 binding when phosphorylated, can no longer be phosphorylated (Fig. 5, Table S1). TASK-1 S392A is expected to recruit 14-3-3 proteins efficiently (Fig. 2, Table 1) and, indeed, we observed equal cell surface expression of the constructs that comprised the exposed TASK-1 S392A or TASK-3 C-termini (Fig. 6).

We also quantitatively compared the effects on 14-3-3 binding for two commonly and interchangeably used manipulations of the TASK-C-termini – mutation of the crucial serine residue in the mode III 14-3-3-binding motif to alanine or deletion of the C-terminal valine. Both variants have been widely used to dissect the role of 14-3-3 in cell surface expression because they have been assumed to impair 14-3-3 binding (O'Kelly et al., 2002; Rajan et al., 2002; Smith et al., 2011). Our results clearly indicate that the two manipulations have different effects on 14-3-3 binding – the mode III serine in its phosphorylated form is indeed an essential pre-requisite for any detectable 14-3-3 binding because 14-3-3 makes many intricate contacts with the phosphate group of the corresponding phosphoserine (Anders et al., 2013), whereas TASK-3 pS373 ΔV374 bound to 14-3-3 with higher affinity than TASK-1 pS393 (Fig. 2, Table 1). These observations indicate that in the context of a phosphorylated mode III binding motif, the C-terminal valine does not contribute substantially to the observed binding affinity, in line with the previously reported structure (Anders et al., 2013), which shows one hydrogen bond between the C-terminal carboxy group of TASK-3 and a lysine residue of 14-3-3σ. However, we also observed that the biological outcome of the two manipulations is very similar for TASK-3 (Fig. 4) because both TASK-3 S373A and TASK-3 ΔV374 failed to reach the cell surface and accumulated in COPI-positive structures (Fig. S1) that partially colocalized with markers of the cis-Golgi (GM130; Fig. 4). Importantly, when investigating the efficiency by which PKA phosphorylates TASK-3 ΔV374, we found that the recombinant catalytic subunit of PKA modifies this construct less efficiently than TASK-3 wild type and that in transfected cells only a fraction of CD8–CFP–TASK-3 ΔV374 was phosphorylated, whereas the corresponding wild-type construct was fully phosphorylated (Fig. 4). From what is known about the PKA consensus sequence (Shabb, 2001), it is expected that a C-terminal serine would be phosphorylated less efficiently, consistent with our results. In conclusion, effects on phosphorylation efficiency have to be considered in addition to effects on COPI or 14-3-3 binding affinity when comparing the *in vivo* outcome of manipulations in the C-terminal TASK trafficking control region. The lower affinity of COPI (Fig. 7) to TASK-1 and of 14-3-3 proteins (Fig. 2, Table 1) to TASK-1 pS393 (as compared to TASK-3 or TASK-3 pS373) allows for a dynamic steady state where phosphatases, kinases and COPI can probe or modify the trafficking control region more often than is the case for TASK-3 (Figs 6B and 7C).

Our discovery that the TASK-1 trafficking control region contains a second serine residue that is inhibitory to 14-3-3 binding when phosphorylated (Fig. 5, Table S1) is reminiscent of a mechanism that has been previously shown to operate on a class of cardiac ATP-sensitive K^+^ channels (K_ATP_) comprising Kir6.2 and SUR1 (Arakel et al., 2014). Both subunits of these channels expose arginine-based COPI recognition signals, which can also recruit 14-3-3 proteins when presented in several copies and close vicinity (Yuan et al., 2003). Phosphorylation by PKA uncouples these peptide-sorting motifs from recognition by COPI, thereby rendering the requirement for 14-3-3 binding obsolete and allowing cell surface expression to be regulated by signal transduction. This might be particularly relevant in cardiac myocytes, which have to adapt their electrical excitability to physiological stimuli and therefore might rely on preassembled ion channels that are stored in the Golgi because COPI retains them there (Arakel et al., 2014). Interestingly, cardiac myocytes also express TASK-1 (Putzke et al., 2007). Hence, the TASK-1 trafficking control region might lead to the accumulation of a Golgi-localized TASK-1 population that can be deployed upon β-adrenergic stimulation and concomitant phosphorylation by PKA, similar to the scenario that has been reported for K_ATP_. In this situation, double phosphorylation of the TASK-1 trafficking control region could trigger COPI- and 14-3-3-independent cell surface expression of a previously synthesized and assembled TASK-1 pool.

In conclusion, our results illustrate how the binding parameters of protein–protein interactions with a trafficking control region of a membrane protein can be poised to enable physiological regulation of cell surface expression. They also underscore the need to study not only the kinases but also the phosphatases that have access to cargo membrane proteins in the secretory pathway of different cell types.

## MATERIALS AND METHODS

### Reagents

Tables detailing antibodies (Table S2), plasmids (Table S3) and primers (Table S4) used in this study are available as supplementary material.

### Molecular biology

GST-conjugated C-termini of TASK-1 and TASK-3, and different mutants were created according to Mant et al. (2011). A detailed list of plasmids and primers can be found in Tables S1 and S2.

Reporter constructs containing the extracellular domain of CD8, a fluorophore (eCFP) and the last 15 amino acids of the TASK-1 and TASK-3 C-termini or different mutated C-termini were created by initial sub-cloning of CD8 from constructs that have been previously described by Zuzarte et al. (2009). The PCR product was digested with *Bam*HI and *Eco*RI, and ligated into the multiple cloning site of pcDNA3.1. eCFP was amplified by using PCR from pECFP-N1 (Clontech, Heidelberg, Germany). PCR products were digested with *Eco*RI and *Not*I, and subsequently ligated into the pcDNA3.1 vector, fusing CD8 and CFP. Oligonucleotides encoding the last 15 amino acids of TASK-1, TASK-3 and the different mutants were annealed and ligated into pcDNA3.1 between *Not*I and *Xba*I restriction sites. Open reading frames encoding human 14-3-3 proteins were inserted into the pMAL2Cx vector at *Eco*RI and *Hind*III restriction sites.

### Purification of proteins

GST proteins fused to the TASK-1 and TASK-3 C-termini were expressed and purified from the *E. coli* strain BL21(pREP4). Protein expression was induced with 1 mM IPTG for 3 h. Cells were subsequently lysed in GST lysis buffer containing 20 mM HEPES pH 6.5, 150 mM KOAc, 5 mM Mg(OAc)_2_, 1 mM EDTA, 1 mM DTT and 1 mM PMSF. Crude cellular lysates were centrifuged at 100,000 ***g*** for 30 min at 4°C. Cleared lysates were incubated for 90 min with equilibrated glutathione Sepharose beads. The bead slurry was transferred to a gravity column and washed with GST lysis buffer. Bound proteins were eluted with GST elution buffer containing 20 mM HEPES pH 9.5, 150 mM KOAc, 5 mM MgOAc, 1 mM EDTA, 1 mM DTT and 15 mM glutathione. Cells expressing MBP-tagged 14-3-3 proteins were lysed in buffer containing 150 mM NaCl, 5 mM MgCl_2_, 50 mM Tris-HCl pH 7.5 (MBP lysis buffer). Further purification steps were executed analogous to the procedure described for GST fusion proteins. Bound proteins were eluted using MBP lysis buffer supplemented with 20 mM d-maltose. To remove the MBP tag, purified proteins were incubated with Factor Xa for 24 h at 4°C. Both GST fusion proteins and processed MBP-tagged proteins were subjected to size exclusion chromatography using an Äkta purifier (GE Healthcare, Chalfont St Giles, UK) equipped with a Superdex75 size exclusion column. Recombinant human PKA catalytic subunit was expressed and purified with an IP20 resin, as described previously (Olsen and Uhler, 1989; Knape et al., 2015).

### Fluorescence polarization measurements

Interactions between 14-3-3 proteins and TASK-1 and TASK-3 C-terminal peptides and variants were investigated using a fluorescence polarization assay (Moll et al., 2006; Muda et al., 2014). Fluorescein-labeled peptides were purchased from Peps4Life Sciences (Heidelberg, Germany). All measurements were performed in buffer containing 20 mM MOPS, 150 mM NaCl, 0.005% (v/v) CHAPS pH 7.0, using a Fusion™ α-FP microtiter plate reader at room temperature in a 384-well microtiter plater (Optiplate 384, black; Packard, Meriden, CT). 14-3-3 proteins were serially diluted from 100 µM to 1 nM and incubated with 10 nM of fluorescently labeled peptide. The fluorescence polarization signal was detected for 2 s at an excitation wavelength of 485 nm with an emission fluorescence polarization filter wavelength of 535 nm and a photomultiplier voltage of 1100. Data were analyzed with GraphPad Prism 6.0 (GraphPad Software, San Diego, CA) by plotting the obtained fluorescence polarization signal in millipolarization units (mPol) against the logarithm of the 14-3-3 protein concentration. The data were then fitted using a monophasic fit.

### Surface plasmon resonance measurements

Binding of 14-3-3 proteins to the C-terminus of TASK-3 was analyzed using surface plasmon resonance. An HC1000m SPR sensor chip was activated with 1-ethyl-3-(3-dimethylaminopropyl)-carbodiimide and N-hydroxysuccinimide (EDC–NHS) according to the manufacturer's instructions (Xantec Biotechnologies, Düsseldorf, Germany). An antibody against GST (Carl Roth, Karlsruhe, Germany; catalog number 3998.1) was coupled at the N-terminus to the activated chip surface at a flow rate of 15 µl/min and a concentration of 30 µg/ml to a surface density of 4000–8000 µRIU (depending on the maximal capacity of each chip and on each batch). The chip surface was subsequently deactivated with a 180-s injection of 1 M ethanolamine pH 8.5, at a flow rate of 30 µl/min. GST fusion proteins were captured at a flow rate of 30 µl/min to a surface density of 200–400 µRIU onto the ligand and reference channel. Proteins captured on the ligand channel were phosphorylated *in vitro* by injecting a phosphorylation buffer containing 20 mM HEPES, 150 mM NaCl, 10 mM MgCl_2_, 100 µM ATP, 200 nM PKA catalytic subunit and 0.005% (v/v) Tween 20 pH 7.5 at a flow rate of 15 µl/min for 20 min. A serial dilution of the analyte [14-3-3 proteins or PKA-phosphorylated-substrate antibody (Cell Signaling Technology, Danvers, MA; clone 100G7E, catalog number 9624) was injected, and association was followed for 4.5 min. Dissociation of the analyte from the ligand was monitored for 7 min. The surface was regenerated after each experiment with glycine pH 2.2. Data analysis was performed using Scrubber 2.0c. Equilibrium binding isotherms were analyzed using GraphPad Prism 6.0. All binding experiments were performed on a Reichert SR 7500DC biosensor instrument at 20°C and a flow rate of 40 µl/min on HC1000m SPR sensor chips (Xantec Bioanalytics, Düsseldorf, Germany) in buffer containing 150 mM NaCl, 20 mM HEPES, 0.005% (v/v) Tween 20, pH 7.5.

### Cell culture and transfection

COS-7 cells were obtained from European Collection of Authenticated Cell Cultures via Sigma-Aldrich (Taufkirchen, Germany; catalog number 87021302) and tested for mycoplasma contamination at regular intervals. For western blotting analysis, COS-7 cells were transiently transfected using the calcium phosphate method. 5 µg of DNA was mixed with 95 µl of 0.25 M CaCl_2_ and 100 µl of BES-buffered saline (BBS; 50 mM BES, 273 mM NaCl, 2 mM Na_2_HPO_4_) and incubated for 30 min at room temperature before adding to cells. Cells were incubated for 20 h at 37°C under 3% CO_2_, washed twice with 1× PBS and then grown for 48 h at 37°C under 5% CO_2_. For indirect immunofluorescence staining, transient transfection was performed on coverslips using FuGENE (Promega, Madison, WI). 150 µl of Opti-MEM (Life Technologies, Karlsruhe, Germany) was mixed with 10 µl of FuGENE and incubated at room temperature for 5 min for equilibration, and added to 5 µg of DNA. After incubation for 15 min at room temperature, 2 ml of Dulbecco's modified Eagle's medium (DMEM; Life Technologies, Karlsruhe, Germany) was added to each tube, and the mixture was transferred to the cells. The cells were incubated with transfection solution for 10 h at 37°C under 5% CO_2_, then washed once with 1× PBS, followed by 38 h of incubation at 37°C under 5% CO_2_.

### *In vitro* phosphorylation assays

GST fusion proteins of the TASK-1 and TASK-3 C-terminus were *in vitro* phosphorylated using recombinant PKA catalytic subunit in 150 mM NaCl, 50 mM Tris-HCl pH 7.5, 10 mM MgCl_2_, 20 nM PKA, 100 µM ATP, as described previously by Mant et al. (2011). Proteins were phosphorylated for either 10 min at room temperature (Fig. 4A,B) or overnight at 4°C. The buffer was supplemented with an ATP regeneration system (Yuan et al., 2003).

### *In vivo* phosphorylation assays and western blotting

1×10^5^ COS-7 cells were transiently transfected using calcium phosphate with 5 µg of DNA and washed twice with 1× PBS, resuspended in 500 µl of membrane preparation buffer [50 mM NaCl, 0.32 M sucrose, 2 mM EDTA, 20 mM HEPES, pH 7.4, cOmplete EDTA-free protease inhibitor cocktail (Roche, Mannheim, Germany) and 50 µM PKA inhibitor H-89 dihydrochloride hydrate (Sigma-Aldrich)], homogenized using a MICCRA-D-1 homogenizer disperser (ART Prozess- & Labortechnik, Müllheim, Germany), followed by 15 strokes with a Dounce homogenizer. After separation of the insoluble fraction with centrifugation for 30 min at 100,000 ***g***, the pellet was solubilized in 250 µl of solubilization buffer (1.5% Triton X-100, 0.75% Na-deoxycholate, 0.1% SDS, 50 mM Tris-HCl, 100 mM NaCl, 5 mM EDTA, 2.5 mM EGTA, pH 7.5) for 30 min on ice, followed by a second centrifugation step. Subsequently, the samples were precipitated with trichloroacetic acid (TCA) and resuspended in λ phosphatase buffer (1× PMP buffer, catalog number P0753, New England Biolabs, Frankfurt, Germany). For each construct, one sample was treated with 1600 U λ phosphatase for 30 min at 30°C, supplemented with 5× SDS loading buffer and separated on 12% SDS gels containing 75 µM Phostag and 75 µM MnCl_2_ reagent. Transfer to a membrane was performed at 4°C for 18 h at 30 V. CD8–CFP–TASK fusion proteins were detected using a CD8-α antibody (Santa Cruz Biotechnology, Santa Cruz, CA; clone H-160, catalog number SC-7188, lot number E2213). Fluorescently labeled secondary antibodies were detected using an Odyssey LiCOR imaging system (LiCOR, Bad Homburg, Germany).

### Quantification of gels

To quantitatively compare the intensity of Coomassie-stained protein bands (Fig. 4A) or signals obtained by detecting specific proteins with western blotting (Fig. 6B), the LiCOR Studio-Lite software was used.

### Flow cytometry

Flow cytometry was performed with a FACS Calibur™ flow cytometer and FACS DIVA™ software (BD Biosciences, Heidelberg, Germany). Cell surface expression of a reporter protein containing the extracellular domain of CD8, an eCFP fluorophore and the last 15 amino acids of the TASK-1 or TASK-3 C-termini, and different mutants was examined using a mouse monoclonal antibody against CD8 (Sigma-Aldrich; catalog number C7423; 5 µl/10^6^ cells in 500 µl PBS) and an Alexa-Fluor-647-coupled secondary antibody (Invitrogen). The ratio between the anti-CD8 antibody staining and the CFP signal was used to determine the relative cell surface expression of constructs bearing different TASK protein C-termini.

### Indirect immunofluorescence staining

Transiently transfected COS-7 cells were fixed 48 h post transfection using a two-step fixation protocol with 2% (w/v) formaldehyde, 0.125 M sucrose in 1× PBS for 20 min at room temperature following fixation with 1% (w/v) formaldehyde in 1× PBS for 10 min. Cells were permeabilized for 10 min at room temperature with 0.3% Triton X-100 and 0.05% SDS in 1× PBS, rinsed twice with PBS and blocked for 30 min at room temperature with 10% (w/v) FBS in 1× PBS. Primary anti-CD8 antibody (Sigma-Aldrich; catalog number C7423, 1:50 dilution), anti-GM130 antibody (Abcam, Cambridge, UK; catalog number ab52649, 1:100 dilution), CD8-α antibody (Santa Cruz Biotechnology, Santa Cruz, CA; clone H-160, dilution 1:200) and anti-βCOP antibody (Sigma-Aldrich; catalog number G6160, dilution 1:2000) were used. Alexa-Fluor-647- or Alexa-Fluor-488-conjugated (Invitrogen) anti-mouse IgG or anti-rabbit IgG secondary antibodies were used at a dilution of 1:500. Coverslips were mounted using Mowiol mounting medium. For Fig. 4D, to stain cell surface-exposed CD8, unfixed cells were washed twice with ice-cold PBS, stained on ice for 30 min with primary antibodies diluted in 5% FBS in 1× PBS, then washed with PBS; the normal fixation protocol was then followed.

### Light microscopy

Wide-field microscopy analysis of fixed and stained cells was performed on a Delta Vision microscope system (GE Healthcare) using a 60×UplanSApo objective. Images were acquired using Coolsnap HQ2 CCDcamera (Roper Scientific), SoftWorx software and were processed using ImageJ software.

### COPI and 14-3-3 pull-down experiments

COPI was purified as described previously (Yip and Walz, 2011). 10 µg of purified GST–Mst27–Task1-C15 or GST–Mst27–Task3-C15 (Sandmann et al., 2003; Michelsen et al., 2007) was phosphorylated by recombinant PKA (phosphorylation buffer: 20 mM HEPES pH 6.8, 2% glycerol, 150 mM potassium acetate, 5 mM magnesium acetate, 1 mM EDTA, 1 mM DTT supplemented with an ATP regeneration system comprising 10 mM phosphocreatine, 0.5 mM ATP, 0.5 mM GTP, 50 µg/ml creatine phosphokinase). Following phosphorylation, the baits were immobilized on ∼20 µl of glutathione–agarose (slurry volume). COPI or purified 14-3-3γ was added as indicated and incubated for a minimum period of 60 min. The baits were washed four times with phosphorylation buffer and eluted with 1× SDS sample buffer (containing 100 mM DTT) and analyzed using SDS-PAGE or Phostag PAGE.

## References

[JCS180182C1] Anders C., Higuchi Y., Koschinsky K., Bartel M., Schumacher B., Thiel P., Nitta H., Preisig-Muller R., Schlichthorl G., Renigunta V. et al. (2013). A semisynthetic fusicoccane stabilizes a protein-protein interaction and enhances the expression of K+ channels at the cell surface. *Chem. Biol.* 20, 583-593. 10.1016/j.chembiol.2013.03.015 23601647

[JCS180182C2] Arakel E. C., Brandenburg S., Uchida K., Zhang H., Lin Y.-W., Kohl T., Schrul B., Sulkin M. S., Efimov I. R., Nichols C. G. et al. (2014). Tuning the electrical properties of the heart by differential trafficking of KATP ion channel complexes. *J. Cell Sci.* 127, 2106-2119. 10.1242/jcs.141440 24569881 PMC4004980

[JCS180182C3] Coblitz B., Shikano S., Wu M., Gabelli S. B., Cockrell L. M., Spieker M., Hanyu Y., Fu H., Amzel L. M. and Li M. (2005). C-terminal recognition by 14-3-3 proteins for surface expression of membrane receptors. *J. Biol. Chem.* 280, 36263-36272. 10.1074/jbc.M507559200 16123035

[JCS180182C4] Kagan A., Melman Y. F., Krumerman A. and McDonald T. V. (2002). 14–3–3 amplifies and prolongs adrenergic stimulation of HERG K+ channel activity. *EMBO J.* 21, 1889-1898. 10.1093/emboj/21.8.1889 11953308 PMC125975

[JCS180182C5] Kilisch M., Lytovchenko O., Schwappach B., Renigunta V. and Daut J. (2015). The role of protein-protein interactions in the intracellular traffic of the potassium channels TASK-1 and TASK-3. *Pflugers Arch.* 467, 1105-1120. 10.1007/s00424-014-1672-2 25559843

[JCS180182C6] Knape M. J., Ahuja L. G., Bertinetti D., Burghardt N. C. G., Zimmermann B., Taylor S. S. and Herberg F. W. (2015). Divalent metal ions Mg^2+^ and Ca^2+^ have distinct effects on protein kinase A activity and regulation. *ACS Chem. Biol.* 10, 2303-2315. 10.1021/acschembio.5b00271 26200257 PMC4714867

[JCS180182C7] Mant A., Elliott D., Eyers P. A. and O'Kelly I. M. (2011). Protein kinase A is central for forward transport of two-pore domain potassium channels K2P3.1 and K2P9.1. *J. Biol. Chem.* 286, 14110-14119. 10.1074/jbc.M110.190702 21357689 PMC3077612

[JCS180182C8] Michelsen K., Schmid V., Metz J., Heusser K., Liebel U., Schwede T., Spang A. and Schwappach B. (2007). Novel cargo-binding site in the beta and delta subunits of coatomer. *J. Cell Biol.* 179, 209-217. 10.1083/jcb.200704142 17954604 PMC2064757

[JCS180182C9] Moll D., Prinz A., Gesellchen F., Drewianka S., Zimmermann B. and Herberg F. W. (2006). Biomolecular interaction analysis in functional proteomics. *J. Neural Transmission* 113, 1015-1032. 10.1007/s00702-006-0515-5 16835689

[JCS180182C10] Muda K., Bertinetti D., Gesellchen F., Hermann J. S., von Zweydorf F., Geerlof A., Jacob A., Ueffing M., Gloeckner C. J. and Herberg F. W. (2014). Parkinson-related LRRK2 mutation R1441C/G/H impairs PKA phosphorylation of LRRK2 and disrupts its interaction with 14-3-3. *Proc. Natl. Acad. Sci. USA* 111, E34-E43. 10.1073/pnas.1312701111 24351927 PMC3890784

[JCS180182C11] O'Kelly I. and Goldstein S. A. (2008). Forward transport of K 2p 3.1: mediation by 14-3-3 and COPI, modulation by p11. *Traffic* 9, 72-78. 10.1111/j.1600-0854.2007.00663.x 17908283

[JCS180182C12] O'Kelly I., Butler M. H., Zilberberg N. and Goldstein S. A. N. (2002). Forward transport. 14-3-3 binding overcomes retention in endoplasmic reticulum by dibasic signals. *Cell* 111, 577-588. 10.1016/S0092-8674(02)01040-1 12437930

[JCS180182C13] Olsen S. R. and Uhler M. D. (1989). Affinity purification of the C alpha and C beta isoforms of the catalytic subunit of cAMP-dependent protein kinase. *J. Biol. Chem.* 264, 18662-18666. 2553718

[JCS180182C14] Putzke C., Wemhoner K., Sachse F. B., Rinne S., Schlichthorl G., Li X. T., Jae L., Eckhardt I., Wischmeyer E., Wulf H. et al. (2007). The acid-sensitive potassium channel TASK-1 in rat cardiac muscle. *Cardiovasc. Res.* 75, 59-68. 10.1016/j.cardiores.2007.02.025 17389142

[JCS180182C15] Rajan S., Preisig-Muller R., Wischmeyer E., Nehring R., Hanley P. J., Renigunta V., Musset B., Schlichthorl G., Derst C., Karschin A. et al. (2002). Interaction with 14-3-3 proteins promotes functional expression of the potassium channels TASK-1 and TASK-3. *J. Physiol.* 545, 13-26. 10.1113/jphysiol.2002.027052 12433946 PMC2290646

[JCS180182C16] Renigunta V., Schlichthorl G. and Daut J. (2015). Much more than a leak: structure and function of K(2)p-channels. *Pflugers Arch.* 467, 867-894. 10.1007/s00424-015-1703-7 25791628

[JCS180182C17] Sandmann T., Herrmann J. M., Dengjel J., Schwarz H. and Spang A. (2003). Suppression of coatomer mutants by a new protein family with COPI and COPII binding motifs in Saccharomyces cerevisiae. *Mol. Biol. Cell* 14, 3097-3113. 10.1091/mbc.E02-11-0736 12925749 PMC181553

[JCS180182C18] Shabb J. B. (2001). Physiological substrates of cAMP-dependent protein kinase. *Chem. Rev.* 101, 2381-2412. 10.1021/cr000236l 11749379

[JCS180182C19] Smith A. J., Daut J. and Schwappach B. (2011). Membrane proteins as 14-3-3 clients in functional regulation and intracellular transport. *Physiology* 26, 181-191. 10.1152/physiol.00042.2010 21670164

[JCS180182C20] Yip C. K. and Walz T. (2011). Molecular structure and flexibility of the yeast coatomer as revealed by electron microscopy. *J. Mol. Biol.* 408, 825-831. 10.1016/j.jmb.2011.03.029 21435344 PMC3096150

[JCS180182C21] Yuan H., Michelsen K. and Schwappach B. (2003). 14-3-3 dimers probe the assembly status of multimeric membrane proteins. *Curr. Biol.* 13, 638-646. 10.1016/S0960-9822(03)00208-2 12699619

[JCS180182C22] Zuzarte M., Heusser K., Renigunta V., Schlichthorl G., Rinne S., Wischmeyer E., Daut J., Schwappach B. and Preisig-Muller R. (2009). Intracellular traffic of the K+ channels TASK-1 and TASK-3: role of N- and C-terminal sorting signals and interaction with 14-3-3 proteins. *J. Physiol.* 587, 929-952. 10.1113/jphysiol.2008.164756 19139046 PMC2673767

